# Supplementary material for: Transition metal binding selectivity in proteins and its correlation with the phylogenomic classification of the cation diffusion facilitator protein family
Source: Sci Rep. 2017 Nov 27;7:16381. doi: 10.1038/s41598-017-16777-5 (PMC5703985; doi:10.1038/s41598-017-16777-5)
Supplement: Supplementary file 1 — ESI1 [file 41598_2017_16777_MOESM1_ESM.doc]

Supplementary Information

**Transition metal binding selectivity in proteins and its correlation with the phylogenomic classification of the cation diffusion facilitator protein family**

**Authors:** Shiran Barber-Zucker^1,2,3^, Boaz Shaanan^1^ and Raz Zarivach^1,2,3^,*

**Authors’** **affiliations:**

^1^ Department of Life Sciences, Ben-Gurion University of the Negev, Beer Sheva 8410501, Israel

^2^ The National Institute for Biotechnology in the Negev, Ben-Gurion University of the Negev, Beer Sheva 8410501, Israel

^3^ Ilse Katz Institute for Nanoscale Science and Technology, Ben-Gurion University of the Negev, Beer Sheva 8410501, Israel

* Correspondence should be addressed to Raz Zarivach, Department of Life Sciences, Ben-Gurion University of the Negev, P.O.B. 653, Beer Sheva 8410501, Israel. Tel: +972-8-6461999; Fax: +972-8-6472970; Email: [zarivach@bgu.ac.il](mailto:zarivach@bgu.ac.il)

**
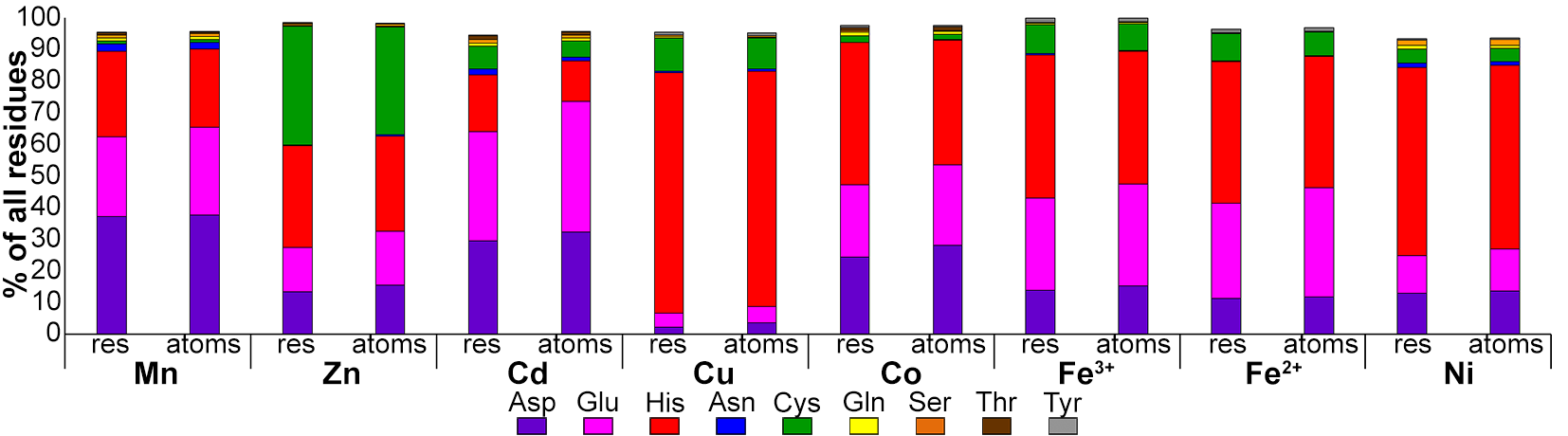
**

**Figure S1.** A comparison between two methods used to analyze amino acids propensities of the different metals shows similar results. In the first approach (termed res, left columns), each residue was counted as one, no matter how many atoms of this residue were bound to the metal, while in the second approach (termed atoms, right columns) each residue was counted as the number of bound atoms from this residue. For every metal, the percentage of each residue is defined as the number of times the specific residue was bound to the metal divided by the number of all residues bound to the metal. The different residues are presented in different colors: Aspartate in purple, glutamate in pink, histidine in red, asparagine in blue, cysteine in green, glutamine in yellow, serine in orange, threonine in brown and tyrosine in gray.


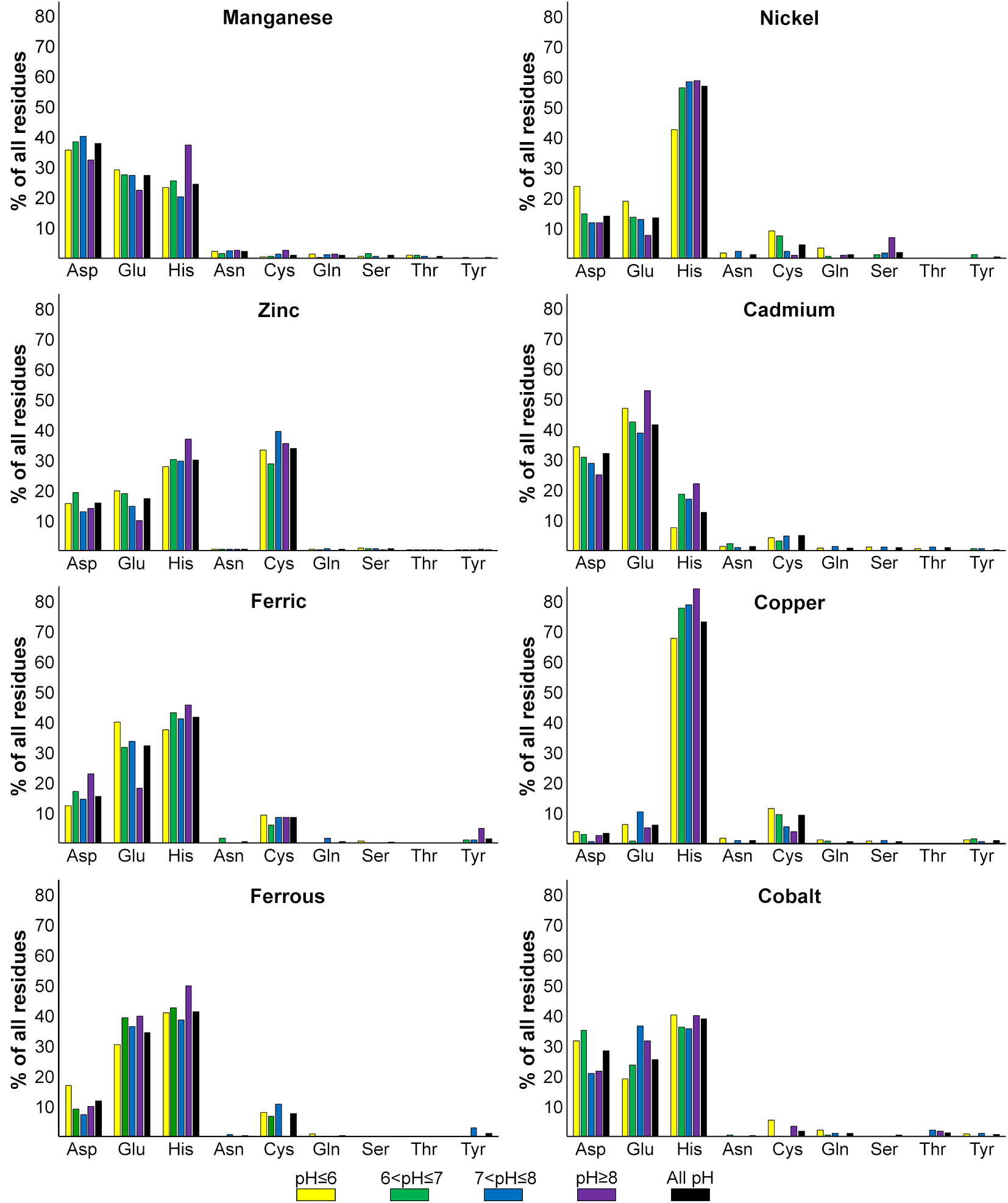


**Figure S2.** pH-dependent analysis of selected amino acid propensities of the different metals. For every metal and pH value, the percentage of each residue is defined as the number of times the specific residue was bound to the metal divided by the number of all residues bound to the metal at the specific pH. The general population was calculated as described in the legend to Figure 3. The different pHs are presented in different colors: pH ≤ 6 in yellow, 6 < pH ≤ 7 in green, 7 < pH ≤ 8 in blue, pH ≥ 8 in purple, while the general population with no pH dependency is shown in black.


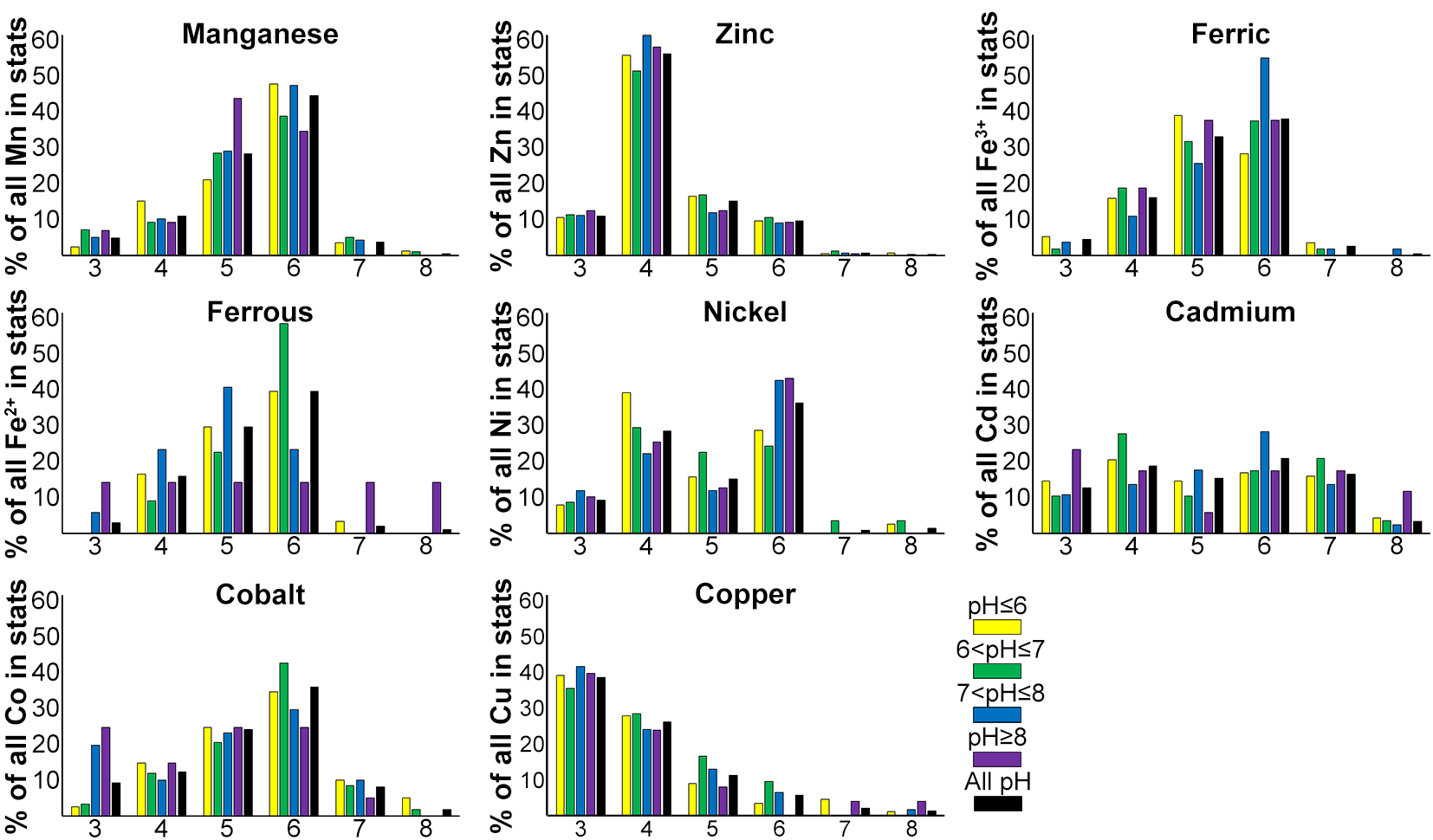


**Figure S3.** pH-dependent coordination number distribution of the different metals. For each metal, the percentage of each coordination number (3 to 8) at each pH value is defined as the number of times the metal was bound in a specific coordination number and pH divided by the total number of times this metal is seen at that pH. The general population was calculated as described in the legend to Figure 4. The different pHs are presented in different colors: pH ≤ 6 in yellow, 6 < pH ≤ 7 in green, 7 < pH ≤ 8 in blue, pH ≥ 8 in purple, while the general population with no pH dependency is shown in black.

**Supplemental Bibliographic References**
